# Surgical antibiotic prophylaxis administration practices

**DOI:** 10.5588/pha.21.0027

**Published:** 2021-11-01

**Authors:** S. Shrestha, K. Hann, K. W. Y. Kyaw, P. Koju, M. Khogali

**Affiliations:** 1 Dhulikhel Hospital, Kathmandu University Hospital, Dhulikhel, Kavre, Nepal; 2 Sustainable Health Systems, Freetown, Sierra Leone; 3 Department of Operational Research, International Union Against Tuberculosis and Lung Disease (The Union), Mandalay, Myanmar; 4 Centre for Operational Research, The Union, Paris, France; 5 UNICEF/UNDP/World Bank/WHO Special Programme for Research and Training in Tropical Diseases (TDR), WHO, Geneva, Switzerland

**Keywords:** antibiotic guidelines, surgical site infections, SORT-IT, AMR

## Abstract

**SETTING:**

A referral hospital in Kavre, Nepal.

**OBJECTIVES:**

To assess 1) compliance with National Antibiotic Treatment Guidelines (NATG), specifically, whether the administration of surgical antibiotic prophylaxis (SAP) (initial dosing and redosing) was in compliance with NATG for patients who were and were not eligible, and 2) development of surgical site infections (SSIs) among patients who underwent surgery in the Department of General Surgery (July–December 2019).

**DESIGN:**

This was a retrospective cohort analysis.

**RESULTS:**

The analysis included 846 patients, of which 717 (85%) patients were eligible for SAP and 129 (15%) were ineligible. Of those eligible, 708 (99%) received the initial dose; while 65 (50%) of the ineligible did not receive any dose. Of those who received the initial dose, 164 (23%) were eligible for redosing. Of these, only 23 (14%) received at least one redosing and 141 (86%) did not receive it. Overall compliance with NATG was achieved in 75% (632/846) of patients. SSIs occurred in 23 (3%) patients, 8 (35%) of whom did not have SAP administered according to NATG.

**CONCLUSION:**

A relatively high overall compliance with NATG for SAP administration was reported. Recommendations were made to improve compliance among those who were ineligible for SAP and those who were eligible for redosing.

## Abstract (FR)

**LIEU:**

Un hôpital de référence du district de Kavre, Népal.

**OBJECTIFS:**

Évaluer 1) le respect des directives nationales sur les traitements antibiotiques (NATG), plus particulièrement si l’administration d’une antibioprophylaxie chirurgicale (SAP) (dose initiale et nouvelle dose) respectait les directives NATG pour les patients qui y étaient ou non éligibles ; et 2) le développement d’infections du site opératoire (SSI) chez les patients ayant subi une intervention chirurgicale dans le service de Chirurgie Générale (juillet–décembre 2019).

**MÉTHODE:**

Il s’agissait d’une analyse de cohorte rétrospective.

**RÉSULTATS:**

L’analyse a inclus 846 patients, dont 717 (85%) étaient éligibles à une SAP et 129 (15%) n’y étaient pas éligibles. Parmi ceux qui y étaient éligibles, 708 (99%) ont reçu la dose initiale, alors que 65 (50%) des patients non éligibles n’ont reçu aucune dose. Parmi ceux ayant reçu la dose initiale, 164 (23%) étaient éligibles à une nouvelle dose. Parmi ces derniers, seuls 23 (14%) ont reçu au moins une nouvelle dose et 141 (86%) n’en ont pas reçu. Les directives NATG ont été respectées chez 75% (632/846) des patients. Des SSI ont été observées chez 23 (3%) patients ; pour huit (35%) de ces patients, la SAP n’avait pas été administrée conformément aux directives NATG.

**CONCLUSION:**

Un respect global relativement élevé des directives NATG pour l’administration de la SAP a été rapporté. Des recommandations ont été émises pour améliorer le respect de ces directives chez les patients non éligibles à la SAP et chez ceux éligibles à une nouvelle dose.

Surgical site infections (SSIs) are infections occurring at the incision site or deep tissue space within 30 days of surgery.^1^ They are one of the most frequent healthcare-associated infections (HAIs) globally, and account for 8% of all associated deaths.^2^ The burden of SSIs is higher in low- and middle-income countries (LMICs), affecting one third of patients who undergo surgery.^3^ Moreover, SSIs lead to increased morbidity, mortality and overall cost of medical care.^4^

Many factors contribute to the occurrence of SSIs; however, contamination of the incision site by pathogenic microbes remains the most established risk factor.^5^ Surgical antibiotic prophylaxis (SAP) refers to the administration of antibiotics to patients in surgical practice.^6^ When appropriately used, SAP can reduce the risk of SSIs and related morbidity and mortality.^7^ However, inappropriate use of SAP leads to the emergence and spread of antibiotic resistance, increases patients’ morbidity, prolongs hospital stays and poses an economic burden on health care.^8^,^9^ Nearly 30–50% of antibiotics prescribed in hospital practice are used for SAP.^10^ However, 40% of prescriptions were found to be inappropriate. This includes, most commonly, wrong choice of antibiotic, administration at the wrong time or continuation of treatment longer than recommended.^11^

Guidelines for antibiotic prophylaxis have been developed worldwide to optimise the use of antibiotics based on available clinical indications and emerging health issues.^12^,^13^ These guidelines are key to ensuring appropriate antibiotic use and are a critical component of antimicrobial stewardship programmes in hospitals.^12^,^14^ However, previous studies have shown that non-compliance with such guidelines remains a challenge, especially in developing countries.^12^,^15^

In 2014, Nepal formulated its National Antibiotic Treatment Guidelines (NATG), which include guidance on SAP (Section IV).^16^ According NATG, patients who undergo surgery, except those with clean wounds (Table 1), must receive an initial dose of SAP intraoperatively, and a repeat dose of SAP (redosing) if the duration of surgery is longer than 2 hours. To date, there has been no formal assessment of compliance with the nationally developed guidance on SAP in Nepal to better understand the current practice of SAP use. Monitoring of compliance can also inform decisions, regulations and interventions to enhance strict implementation of existing guidelines and promote prudent antibiotic use in hospital settings.^17^

**TABLE 1:** Surgical wound class definition and indication (eligibility) for SAP according to Nepal National Antibiotic Treatment Guidelines^16^

Wound class	Definition	Indication for SAP
Clean	Primarily closed, elective procedures involving no inflammation, no break in technique, and no entry into the gastrointestinal, oropharyngeal, biliary, genitourinary tracts or tracheobronchial tracts (e.g., herniorrhaphy)	Not recommended Recommended only if: 1) insertion of prosthesis during surgery; or 2) patient has a comorbidity
Clean-contaminated	Surgery during which colonised viscus (e.g., gastrointestinal, tracheobronchial or genitourinary tract) is entered; minor breaches in technique; procedures following blunt trauma; cholecystectomy; prostate surgery; upper and/or lower urinary tract surgery; or uncomplicated appendectomy	Recommended
Contaminated	Surgery in the presence of non-purulent inflammation or major spillage from a colonised viscus, major breach in aseptic technique, or traumatic wounds less than 4 hours old	Recommended
Dirty	Surgery in the presence of established infection (e.g., perforated viscous, devitalised tissue) and traumatic wounds more than 4 hours old	NA^*^

^*^ As dirty wounds are provided therapeutic antibiotics before surgery, these do not qualify for SAP.

SAP = surgical antibiotic prophylaxis; NA = not applicable.

The aim of the present study was to assess compliance with the NATG for administration of SAP in patients who underwent surgery in the Department of General Surgery at Dhulikhel Hospital in Kavre, Nepal. Specific objectives were to report on 1) demographic and clinical characteristics; 2) compliance with NATG (2014), specifically, whether the administration of SAP (initial dosing and redosing) was in compliance with NATG for the patients who were and were not eligible, and 3) development of SSIs among all patients who underwent surgery between July and December 2019.

## METHODS

### Study design

This was a retrospective cohort study.

### Study setting

Nepal is a low-income country in South-East Asia, bordering India in the east, west and south and China in the north. Its population size is about 30 million people, of which 21% reside in urban areas.^18^,^19^ As in many other LMICs, antimicrobial resistance (AMR) is one of the major health challenges in the country.^20^

### National Antibiotic Treatment Guidelines (NATG)

The NATG provides guidance on how to administer SAP in Section IV.^16^ According to the guidelines, SAP should be administered for clean-contaminated wounds, contaminated wounds, and clean wounds only either when prostheses are inserted during surgery in patients or when patients have comorbidities. Other clean wounds are ineligible for SAP (Table 1).

The guidelines do not include a recommendation about the choice of specific antibiotic to be used for SAP. However, they recommend the use of antibiotics against organisms that are most likely to cause infection. The guidelines do not provide instructions on how to determine the required dose for SAP, nor do the guidelines specify which patients should receive more than one redosing. In addition, surgeons do not receive specific training on how to apply these guidelines.

### Study site

Dhulikhel Hospital is a referral hospital located 30 km east of Kathmandu. The hospital has 425 beds, which are distributed across 10 departments. The hospital performs 20 surgical operations per day, of which eight operations on average are from the Department of General Surgery. SSIs are diagnosed based on clinical symptoms.

### Study population

All patients who underwent surgery and were admitted in the Department of General Surgery at Dhulikhel Hospital between July and December 2019 were included in the study. Patients with dirty wounds were started on therapeutic antibiotics prior to surgery, and were thus excluded from the analysis related to compliance with NATG. Some patients who had to return to the operating theatre during admission were excluded from the study, as they had been assigned duplicate registration numbers during data entry.

### Data collection and validation

Data on age, sex, anatomical site of surgery, comorbidities, and type and duration of surgery were extracted from the operating theatre record using Microsoft Access (Microsoft, Redmond, WA, USA) by the principal investigator. Data on comorbidities reflect those recorded in the medical records of the patients. Information on surgical wound class and administration of SAP was collected from the medical records and occurrence of SSIs from the SSIs surveillance records by a nurse who was trained in data collection.

Data were double-entered into EpiData v3.1 (Epi-Data Association, Odense, Denmark). The two files were then validated, and discordances were resolved by referring to the original data source.

### Data analysis and statistics

Data were analysed using EpiData Analysis v2.2.2.183. Numbers and proportions were calculated to describe the clinical and demographic characteristics of all patients, administration of SAP and occurrence of SSIs.

For the purpose of this study, compliance is defined according to eligibility for SAP as shown in Table 2.

**TABLE 2:** Definitions of different levels of compliance with the National Antibiotic Treatment Guidelines in SAP administration

Level	Definition
Overall compliance	Fulfilment of the below four levels
Compliance among patients:	
Eligible for initial dose	Administration of initial dose of SAP at the correct time
Ineligible for initial dose	Non-administration of any dose of SAP
Eligible for redosing of SAP	Administration of redosing
Ineligible for redosing of SAP	Non-administration of redosing

SAP = surgical antibiotic prophylaxis.

### Ethics approval

Ethics approval was obtained from Institutional Review Committee of Kathmandu University School of Medical Sciences, Kathmandu, Nepal (IRC 20/20) and from the Union Ethics Advisory Group of the Center for Operational Research at the International Union Against Tuberculosis and Lung Disease, Paris, France (EAG 10/20).

## RESULTS

### Demographic and clinical characteristics of surgical patients

Between July and December 2019, 874 patients underwent surgery in the Department of General Surgery. Table 3 shows the demographic and clinical characteristics of these patients. The majority of patients (*n* = 497, 57%) were male. The median age of patients was 40 years (interquartile range [IQR] 26–53). Of all anatomical sites of surgery involved, the most common were gastrointestinal (*n* = 476, 54%), followed by inguinal hernia (*n* = 128, 15%) and the upper urinary site (*n* = 101, 12%). The most common type of surgery was elective (*n* = 661, 76%). Surgical wounds were classified as clean-contaminated (*n* = 587, 67%), clean (*n* = 202, 23%), contaminated (*n* = 57, 7%) and dirty (*n* = 28, 3%). Comorbidity was reported in only 55 (6%) patients, with diabetes mellitus being the most common (*n* = 31, 4%). Prostheses were inserted in 13% (*n* = 116) of patients. (Table 3)

**TABLE 3:** Demographic and clinical characteristics of patients who underwent surgery in the Department of General Surgery at Dhulikhel Hospital, Kavre, Nepal, July–December 2019

Characteristics	Patients who underwent surgery	

	*n*	(%)
Total	874	(100)
Sex		
Male	497	(57)
Female	377	(43)
Age, years		
0–10	74	(8)
11–20	84	(10)
21–40	310	(35)
41–60	294	(34)
⩾61	112	(13)
Median [IQR]	40	[26–53]
Anatomical site of surgery		
Gastrointestinal	476	(54)
Inguinal hernia	128	(15)
Upper urinary	101	(12)
Lower urinary	54	(6)
Thoracic	8	(1)
Vascular	42	(5)
Others	65	(7)
Type of surgery		
Elective	661	(76)
Emergency	213	(24)
Surgical wound class		
Clean	202	(23)
Clean-contaminated	587	(67)
Contaminated	57	(7)
Dirty	28	(3)
Comorbidity		
Cancer	16	(2)
TB	8	(1)
Diabetes mellitus	31	(3)
None	819	(94)
Insertion of prosthesis		
No	758	(87)
Yes	116	(13)
Duration of surgery, h		
⩽2	673	(80)
>2	173	(20)

IQR = interquartile range.

### Administration of surgical antibiotic prophylaxis and occurrence of surgical site infections

Overall compliance with the NATG for SAP administration was observed in 75% (632/846) of all patients included in the analysis. The administration of SAP and occurrence of SSIs are shown in the Figure. Of the 846 patients included in the analysis, 717 (85%) were eligible for the initial dose of SAP, and 129 (15%) with clean wounds were not eligible for any dose of SAP. Of those eligible for the initial dose of SAP, 708 (99%) patients received it and all (100%) received them at the correct time. Only 9 (1%) patients of those eligible did not receive any dose of SAP. Of those not eligible for SAP, 64 (50%) received the initial dose. Of those who received the initial dose of SAP according to NATG, 164 (23%) were eligible for redosing, 141 (86%) of whom did not receive redosing.

**FIGURE:**
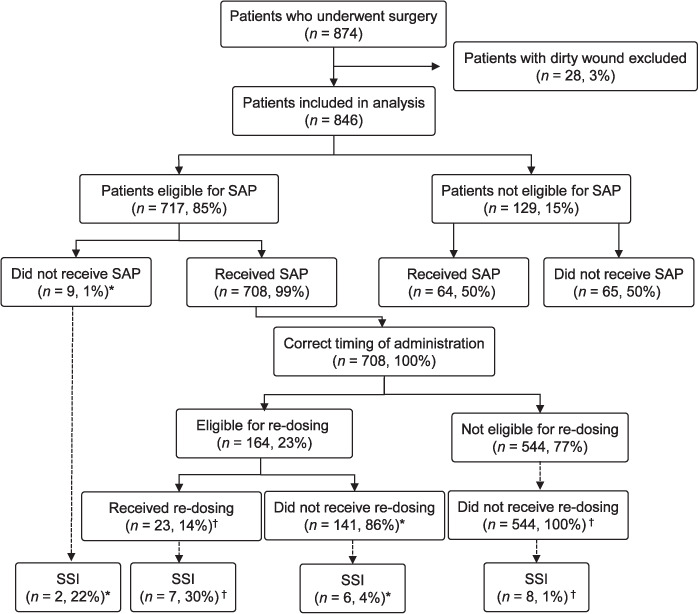
Administration of SAP and occurrence of SSIs among patients who underwent surgery and were admitted to the Department of General Surgery at Dhulikhel Hospital, Kavre, Nepal, July–December 2019. ^*^Patients whose SAP was not administered in compliance with NATG and developed SSI. ^†^Patients whose SAP was administered in compliance with NATG and devloped SSI. SAP = surgical antibiotic prophylaxis; SSI = surgical site infection; NATG = National Antibiotic Treatment Guidelines.

Of all patients included in the analysis, SSIs occurred in 23 (3%) patients. Of these, 2 (22%) were eligible for SAP, but did not receive any dose; 7 (30%) were eligible for and received it; 6 (4%) were eligible for redosing and did not receive it; and 8 (1%) were not eligible for redosing. Of all patients who developed SSIs, SAP was administered in compliance with NATG in 15 (65%). Among eligible patients who were administered SAP in compliance with NATG, 15/567 (2.6%) developed SSI, and among eligible patients who were not administered SAP in compliance with NATG, 8/150 (5.3%) developed SSI.

## DISCUSSION

This is the first study to assess the practice of administering SAP in accordance with NATG among patients who undergo surgery in Nepal. The study showed that administration of SAP was according to guidelines in 75% of patients. This is considered a high compliance rate compared to the 21% reported in a study conducted by Shankar et al. in Nepal prior to the development of NATG.^21^ Shankar et al. assessed the appropriateness of administering SAP against the American Association of Hospital Pharmacists (2007) guidelines.^21^ The high rate of compliance observed in our study can be explained by the fact that the national guidelines were locally adapted, more accessible to users, and took into consideration available drugs and infrastructure, and the skills of the healthcare providers.^22^

Our study had several strengths. First, we used routinely collected data; thus, findings are likely to reflect the operational reality on the ground. Second, the data collector was well trained and supervised by the principal investigator, which ensured data quality. Third, we followed standard definitions and classifications for eligibility, which allowed for comparing our findings with other studies; and fourth, we followed the Strengthening the Reporting of Observational Studies in Epidemiology guidelines for reporting observational studies.^23^

The study limitations included the following: 1) there was no information on the choice and dose of SAP in NATG and, thus, these were not part of the criteria for assessing compliance with the national guideline in our study; and 2) a lack of explanation for several findings in our study. We were not able to determine the reasons for administering SAP in ineligible patients, nor the reasons why those who were eligible for redosing did not receive it. However, for those who were eligible for the initial dose of SAP and did not receive it and those who were eligible for redosing and did not receive it, may have been the lack of training of the surgeons, which may affect eligibility for SAP. This situation could be influenced by several factors, including lack of knowledge and skills,^24^ poor awareness of AMR and strong beliefs relating to low levels of contamination.^25^

Despite these limitations, our study revealed some interesting findings, which warrant further discussion. First, 50% of those who were not eligible for SAP administration, received it. This rate is higher than that has been reported in other studies.^26^,^27^ This practice potentially diverts antibiotics from those who may be in need of them. Furthermore, this practice also falls under irrational use of antibiotics, which further contributes to the problem of AMR in Nepal.^8^,^9^ This points to the need for the hospital management team to address training on NATG and implement efforts towards antibiotic stewardship, as well as further investigations to understand reasons for such practice.

Second, 99% of those eligible for SAP in this study, received the initial dose. This is an encouraging finding and much higher than that reported by Parulekar et al. in India (68%).^28^ However, 1% of those eligible did not receive SAP. Efforts should be made to ensure that SAP is administered in all those who are eligible for it, including by emphasising eligibility criteria during training and stewardship programmes.

Third, SAP was administered at the correct time in all patients who received the initial dose. Studies have reported lower rates of correct timing for the initial dose,^29^,^30^ including in India (89%) and Pakistan (40%).^28^,^31^ This high rate can be attributed to the fact that correct timing of SAP administration is also re-emphasised by the infection prevention and control (IPC) manual (Chapter on HAIs) in the hospital.^32^

Fourth, SAP was not administered in almost nine out of every 10 patients who were eligible for redosing. This means that these patients received a sub-optimal dose of SAP, and were thus at increased risk of SSIs occurrence.^10^,^11^ This may indicate the need for the hospital management to develop a training programme for surgeons on NATG, which specifically references the importance of redosing in SAP.

Fifth, SSIs occurred in three of every 10 patients who received the initial dose and redosing of SAP according to NATG. This implies that SAP failed to prevent all SSIs and may be due to the fact that the choice of antibiotic, dose and frequency of redosing of SAP were left to the discrimination of the operating surgeon. As such, surgeons might have wrongly calculated the required dose of SAP, missed the required frequency of redosing or selected antibiotics for which resistance had already developed. There is a need to update the existing NATG with clear direction on the choice of antibiotics that can be used for SAP, how to calculate the required dose according to the patient’s bodyweight, and to specify the number and frequency of additional redosing required according to the duration of surgery.

Sixth, among those who were eligible for redosing, the rate of SSI occurrence was almost eight times higher in those who received it than those who did not. This is contrary to what has been reported by Miliani et al.^33^ As surgeons are not trained on NATG, decisions for selecting patients for redosing could be based on an alternative clinical indicator, which is not represented in our data; thus, this finding could represent a selection bias for patients at higher risk for SSIs. Nevertheless, this finding warrants further investigation.

Finally, an important operational finding is that the guidance on SAP administration is embedded within broader guidelines for antibiotic treatment. This might create apathy among surgeons to read the full guidelines, and, thus, the section on SAP might be overlooked. The hospital management team in Dhulikhel Hospital should ensure that there is a separate protocol for SAP administration and that all operating surgeons are trained in those protocols.^24^ This is an important step in building an antimicrobial stewardship programme in the hospital. Furthermore, surgeons must be involved in the development of these protocols to build the sense of ownership and enhance the likelihood of protocol compliance.^34^

In conclusion, our study showed a relatively high rate of overall compliance with NATG for SAP administration compared to rates reported by other studies. However, we identified key considerations related to compliance with the guidelines among patients ineligible for initial dose of SAP and those eligible for redosing. Antibiotics were administered in 50% of patients who were ineligible for SAP; 1% of those were eligible for SAP did not receive any dose; and 86% of those were eligible for redosing did not receive it. Our study has also identified several guidelines shortcomings related to the choice of antibiotics, dose and frequency of redosing of SAP. Recommendations were made to the hospital management team at Dhulikhel Hospital to address each of these gaps and strengthen AMR stewardship in the hospital.
